# Genetic and biological characterization of H9N2 avian influenza viruses isolated in China from 2011 to 2014

**DOI:** 10.1371/journal.pone.0199260

**Published:** 2018-07-03

**Authors:** Rui Zhu, Danwen Xu, Xueqin Yang, Jianjun Zhang, Shifeng Wang, Huoying Shi, Xiufan Liu

**Affiliations:** 1 College of Veterinary Medicine, Yangzhou University, Yangzhou, Jiangsu, PR China; 2 Jiangsu Co-innovation Center for the Prevention and Control of Important Animal Infectious Diseases and Zoonoses, Yangzhou, PR China; 3 Sinopharm Yangzhou VAC Biological Engineering Co., Ltd., Yangzhou, Jiangsu, PR China; 4 Department of Infectious Diseases and Pathology, College of Veterinary Medicine, University of Florida, Gainesville, Florida, United States of America; Icahn School of Medicine at Mount Sinai, UNITED STATES

## Abstract

The genotypes of the H9N2 avian influenza viruses have changed since 2013 when almost all H9N2 viruses circulating in chickens in China were genotype 57 (G57) with the fittest lineage of each gene. To characterize the H9N2 variant viruses from 2011 to 2014, 28 H9N2 influenza viruses were isolated from live poultry markets in China from 2011–2014 and were analyzed by genetic and biological characterization. Our findings showed that 16 residues that changed antigenicity, two potential N-linked glycosylation sites, and one amino acid in the receptor binding site of the HA protein changed significantly from 2011–2014. Moreover, the HA and NA genes in the phylogenetic tree were mainly clustered into two independent branches, A and B, based on the year of isolation. H9N2 virus internal genes were related to those from the human-infected avian influenza viruses H5N1, H7N9, and H10N8. In particular, the NS gene in the phylogenetic tree revealed genetic divergence of the virus gene into three branches labeled A, B, and C, which were related to the H9N2, H10N8, and H7N9 viruses, respectively. Additionally, the isolates also showed varying levels of infection and airborne transmission. These results indicated that the H9N2 virus had undergone an adaptive evolution and variation from 2011–2014.

## Introduction

The subtype H9N2 low-pathogenic avian influenza viruses have been prevalent in China since 1994. H9N2 viruses can cause great economic losses to the domestic poultry industry when co-infected with H5 or H7 influenza viruses or other pathogens including *Escherichia coli*, *Chlamydia psittaci*, *Ornithobacterium rhinotracheale*, *Staphylococcus aureus*, *Haemophilus paragallinarum* and others [1–5]. Aside from waterfowl and poultry, different mammalian hosts, including humans, have been infected with H9N2 influenza viruses [6–9]. Additionally, H9N2 viruses also contribute to the reassortment of other virus subtypes, such as the human-infected H5N1 and H7N9 viruses and the emerging avian influenza viruses H6N1 and H10N8 [10–14]. Therefore, H9N2 viruses have become a potential threat to public health.

Hemagglutinin (HA) is an important surface protein that plays a vital role when influenza viruses are introduced into host cells. According to epidemiological and genetic studies, the HA genes in H9N2 viruses can be divided into three distinct lineages in China including A/chicken/Beijing/1/94-like (H9N2, BJ/94-like), A/quail/Hong Kong/G1/97-like (H9N2, G1-like), and A/duck/Hong Kong/Y439/97-like (H9N2, Y439/97-like) [15]. Since 2010, most H9N2 isolates have clustered into genotype 57 (G57) in the BJ/94-like lineage. G57 strains have been predominant in China since 2013 [16]. It has been recently reported that approximately 18 residues are antigenically relevant: 66, 90, 127, 145, 153, 164, 167, 168, 181, 198, 200, 201, 216, 220, 235, 254, 256, 283. These are present in the HA protein from the H9 subtype avian influenza virus, almost all of which are located in the HA globular domain [15, 17–19]. The antigenic residues in H9 are scattered throughout the globular domain instead of the five typical antigenic epitopes in H3 HA [18]. The H9N2 virus has undergone significantly antigenic drift over the past two decades, and the three commercial H9N2 vaccine strains isolated in the 1990s A/chicken/Shanghai/F/98 (H9N2, SH/F/98), A/chicken/Shandong/6/96 (H9N2, SD/6/96) and A/chicken/Guangdong/SS/94 (H9N2, GD/SS/94) cannot effectively protect poultry from the current field strains [20–22].

To understand the evolutionary characteristics of the H9N2 avian influenza viruses from 2010–2014, molecular changes, the antigenic residues, potential glycosylation sites, and receptor-binding sites in HA from 28 H9N2 strains isolated from 2011 to 2014 were analyzed. Moreover, the infection and airborne transmission from eight H9N2 isolates in this study were identified.

## Materials and methods

### Ethics statement

The specific-pathogen-free (SPF) chickens and chicken embryos used in this study were purchased from Beijing Center for Laboratory Animals. Procedures involving the care and use of animals were approved by the Jiangsu Administrative Committee for Laboratory Animals (permission number SYXK-SU-2007-0005) and complied with the Jiangsu Laboratory Animal Welfare and Ethics guidelines of the Jiangsu Administrative Committee of Laboratory Animals.

### Sample collection and viral isolation

From 2011 to 2014, tracheal or cloacal swabs collected from live poultry markets in China, including Jiangsu, Anhui, Heilongjiang, and Fujian, were placed into phosphate buffered saline (PBS) containing 2000 units/mL penicillin, 2 mg/mL streptomycin, 50 μg/mL gentamycin, and 1000 units/mL mycostatin. After freezing, the supernatants were collected through a 0.22 μm pore size filter after centrifugation at 1000 g for 10 min at 4°C. Next, 200 μL of the supernatant was inoculated in the allantoic cavities of 10-day-old SPF embryonated chicken eggs. After 72 h of incubation at 37°C, the viruses were tested for HA activity. The isolated viruses were identified as H9 subtype by hemagglutination inhibition (HI) assay using antisera against H9 subtype, H5 subtype avian influenza viruses (AIV), and Newcastle disease viruses (NDV). The 50% egg infective doses (EID_50_) for the isolates were determined by propagating serial dilutions of viruses in eggs (0.2 mL per embryo) and calculated as described by Reed & Muench [23]. The isolate HI assays were performed with an initial dilution of 1:2 as previously described [24].

### Genetic and phylogenetic analysis

Total viral RNA was extracted from allantoic fluids infected with purified isolates using the TRIZol Reagent (Invitrogen Life Technologies, Carlsbad, CA, USA). Viral RNA was reverse-transcribed (PrimeScript**II**Reverse Transcriptase, Takara Biotechnology, Dalian, China) into cDNA using 12-base universal primers for influenza A viruses (U12 A/G: AGCG/AAAAGCAGG). The gene fragments were amplified by polymerase chain reaction (PrimeSTAR Max DNA Polymerase, Takara Biotechnology, Dalian, China) after reverse transcription using specific primers, sequenced, and assembled by Takara Biotechnology (Dalian, China) [25, 26]. The sequences were edited using the Lasergene software package (DNASTRA Inc., Madison, WI, USA). Phylogenetic trees were generated using the MEGA6.0 software suite. The neighbor-joining method with 1000 bootstrap replicates was performed for the phylogenetic and molecular evolutionary analysis. The phylogenetic lineages of all eight genes were defined by gene phylogeny as previously described [26].

### Key site analysis

To investigate the antigenic drift, the mutation levels for each of 18 reported antigenic residues from 28 isolates were quantified by comparison with 1081 full-length H9N2 HA sequences deposited in GenBank from 2010 to 2015 (date to Jan 1, 2016). The three commercial vaccine strains SH/F/98, SD/6/96, and GD/SS/94 were used as controls.

The potential N-linked glycosylation sites (PNLGSs) in HA were predicted using the NetNGlyc 1.0 Server online software (http://www.cbs.dtu.dk/services/NetNGlyc/). Some key residues from all gene fragments were also investigated.

### Antigenic analysis of the isolates

To investigate the antigenic relationship between the H9N2 isolates and two commonly used vaccine strains (SH/F/98 and GD/SS/94), the nine H9N2 isolates (SD/C9QH/11, JS/JT12/11, JS/YZ640/12, JS/YZ618/12, JS/TM58/13, JS/TM59/13, JS/JT95/13, AH/WB/14 and JS/TM71/1) and two commercial vaccine strains (SH/F/98 and GD/SS/94) were selected according to the genotype of the isolates and the year of isolation. The antiserum of each strain was made, then the HI titer of each straun were detected as following description. Briefly, 3-week-old SPF chickens were immunized twice by subcutaneous injection of 0.3 mL of oil-emulsion of inactive whole virus vaccine of the indicated virus, which was inactivated by adding 0.2% formalin (*v*/v) for 24h at 37°C. The antisera were collected from vaccinated SPF chickens in two weeks after the booster vaccination and used to characterize the antigenicity of the 28 H9N2 isolates and the two commercial vaccine strains. The HI assay was expressed as the reciprocal of the highest serum dilution in which HA was inhibited. Then, antigenic cartography was performed with the Antigen Map program (http://sysbio.cvm.msstate.edu/AntigenMap), which uses matrix completion multidimensional scaling to map HI titers in two dimensions [27].

### Replication and transmission of H9N2 influenza virus isolates in chickens

To study the viral replication and pathogenicity of the H9N2 influenza virus in chicken, groups of six 3-week-old SPF chickens were orally, intranasally, or intratracheally inoculated with 0.2 mL of 10^6^ EID_50_ of each virus or a PBS control. Tissue samples (trachea and lung) from the inoculated chickens were collected at 3 days and 5 days post-inoculation (dpi). Briefly, three SPF chickens were euthanatized using CO_2_ asphyxiation at designated times, and half of the tissues were harvested, washed, and ground into 20% (*w*/v) suspension in 1 mL PBS. Virus titers in the trachea and lung were determined in 10-day-old SPF embryonated chicken eggs. The other half of the tissues were fixed with 10% neutral buffered formalin, embedded in paraffin, sectioned at 5 mm, and processed for staining with hematoxylin and eosin for histopathological examination. These experiments were repeated three times.

For studying the viral transmission, fifteen 3-week-old SPF chickens were divided into five groups: (i) inoculated group (three chickens), (ii) direct contact group (three chickens), (iii) airborne contact group (three chickens), (iv) PBS control group (three chickens), (v) inoculated group for sera (three chickens). To prevent cross-contamination, the chickens from the experimental groups (inoculated group and direct contact group were in the same poultry isolator) were housed in a separated poultry isolator after fumigation with the formaldehyde-potassium permanganate. The infected group was inoculated orally, intranasally, and intratracheally with 10^6^ EID_50_ of the indicated virus. The airborne contact group was placed in a poultry isolator adjacent to the infected group with a distance of 50 cm between poultry isolators. At day 3 and 5 post-inoculation, tracheal and cloacal swabs from chickens were collected in 1 mL of PBS containing antibiotics and, following one freeze-thaw cycle, were centrifuged at 3000 rpm for 10 min. Of the resulting supernatant, 0.2 mL were taken, and the EID_50_ titers of the tracheal and cloacal swabs collected from the indicated passaged virus were determined by serial titration of viruses in SPF embryonated chicken eggs using the Reed & Muench method [23]. Sera collected from each experimental bird at 21 dpi were tested by HI assay to check the antibody in the serum against the indicated viruses [28]. The experiment was repeated three times.

## Results

### Viral isolation and identification

Allantoic fluids infected with tracheal or cloacal swabs taken from chickens were harvested from embryonated SPF chicken eggs. The viruses were identified as H9 subtype by HI assay using chicken H9 antiserum and were identified as N2 via PCR using specific primers. The isolated viruses did not react with H5 or Newcastle disease virus antiserum. Twenty-eight H9N2 strains from chickens in live poultry markets were isolated in this study. HA genes from the 28 H9N2 isolates and seven other genes (genes neuraminidase (NA), polymerase basic 2 (PB2), polymerase basic 1 (PB1), polymerase acidic (PA), nucleoprotein (NP), matrix (M), nonstructural (NS)) from the 9 representative isolates for the 28 H9N2 isolates were sequenced and submitted to GenBank under the accession numbers MG280611-MG280630 and MG280639-MG280709 (Table 1).

**Table 1:** H9N2 influenza A viruses isolated from live poultry markets.

Viruses	Abbreviated name	Isolation date	Location of isolation	Accession no.
A/chicken/Heiilongjiang/DD/2011	HLJ/DD/11	03/2011	Heilongjiang	MG280611
A/chicken/FuJian/PT/2011	FJ/PT11	03/2011	Fujian	MG280612
A/chicken/Jiangsu/JT12/2011	JS/JT12/11	03/2011	Changzhou	MG280613-MG280620
A/chicken/HeBei/BD/2011	HB/BD/11	04/2011	Hebei	MG280621
A/chicken/Shandong/C9QH/2011	SD/C9QH/11	04/2011	Qihe	MG280622-MG280629
A/chicken/Heilongjiang/DLH/2011	HLJ/DLH/11	05/2011	Heilongjiang	MG280630
A/chicken/Jiangsu/YZ918/2011	JS/YZ918/11	09/2011	Yangzhou	MG280639
A/chicken/Jiangsu/WJ37/2011	JS/WJ37/11	11/2011	Changzhou	MG280640
A/chicken//Jiangsu/YZ150/2012	JS/YZ150/12	02/2012	Yangzhou	MG280641
A/chicken/Jiangsu/YZ177/2012	JS/YZ177/12	02/2012	Yangzhou	MG280642
A/chicken/Jiangsu/YZ253/2012	JS/YZ253/12	03/2012	Yangzhou	MG280643
A/chicken/Jiangsu/YZ562/2012	JS/YZ562/12	04/2012	Yangzhou	MG280644
A/chicken/Zhejiang/618/2012	ZJ/618/12	04/2012	Zhenjiang	MG280645-MG280652
A/chicken/Jiangsu/YZ640/2012	JS/YZ/640/12	04/2012	Yangzhou	MG280653-MG280660
A/chicken/Jiangsu/YZ667/2012	JS/YZ667/12	04/2012	Yangzhou	MG280661
A/chicken/Jiangsu/YZ687/2012	JS/YZ687/12	04/2012	Yangzhou	MG280662
A/chicken/Jiangsu/YZ709/2012	JS/YZ709/12	09/2012	Yangzhou	MG280663
A/chicken/Jiangsu/YZ737/2012	JS/YZ737/12	07/2012	Yangzhou	MG280664
A/chicken/Jiangsu/YZ752/2012	JS/YZ752/12	10/2012	Yangzhou	MG280665
A/chicken/Jiangsu/TM59/2013	JS/TM59/13	08/2013	Changzhou	MG280666-MG280673
A/chicken/Fujian/SN01/2013	FJ/SN01/13	09/2013	Fujian	MG280674
A/chicken/Jiangsu/YZ186/2013	JS/YZ186/13	09/2013	Yangzhou	MG280675
A/chicken/Jiangsu/TM58/2013	JS/TM58/13	10/2013	Changzhou	MG280676-MG280683
A/chicken/Jiangsu/JT95/2013	JS/JT95/13	11/2013	Changzhou	MG280684-MG280691
A/chicken/Jiangsu/TM71/2014	JS/TM71/14	01/2014	Changzhou	MG280692-MG280699
A/chicken/Anhui/WB/2014	AH/WB/14	06/2014	Anhui	MG280700-MG280707
A/chicken/Fujian/SN02/2014	FJ/SN02/14	07/2014	Fujian	MG280708
A/chicken/Jiangsu/MH17/2014	JS/MH17/14	08/2014	Changzhou	MG280709

Abbreviations: HLJ, Heilongjiang; FJ, Fujian; JS, Jiangsu; HB, Hebei; SD, Shandong; ZJ, Zhejiang; and AH, Anhui.

### Phylogenetic analysis

**Fig 1:**
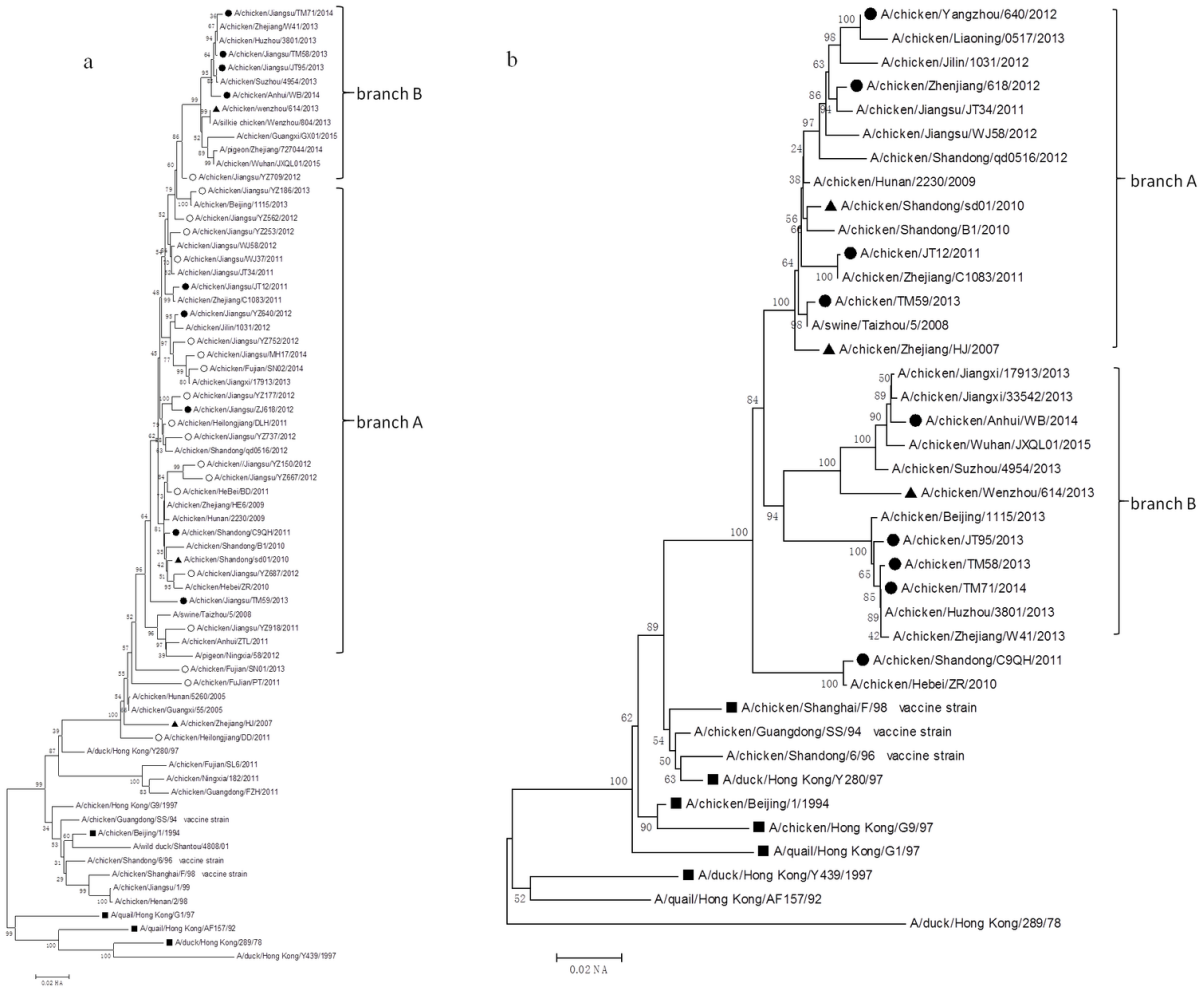
Phylogenetic trees for HA and NA genes collected from mainland China between 2011 and 2014. Solid squares indicate representative strains, solid circles indicate the strains isolated in this study (black circles indicate representative isolates which emerge in the phylogenetic trees for all of the genes except for HA, and white circles indicate the remaining 28 isolates in this study which only emerge in the HA phylogenetic tree), and solid triangles indicate G57 representative strains. Unrooted phylogenetic trees were generated by the distance-based neighbor-joining method using the MEGA6.0 software suite (for the following nucleotide positions: a) HA, 128–1540; b) NA, 87–1263. The percentage of replicate trees in which the associated taxa clustered together in the bootstrap test with 1000 replicates is shown next to the branches.

The phylogenetic analysis included the HA nucleotide sequences from the 28 field isolates and the other 42 reference strains, which include 18 reference strains from different lineages, three vaccine strains, three G57 reference strains, and 18 field strains isolated from 2010–2015. The results showed that HA genes from all 28 H9N2 isolates were clustered into the BJ/94-like lineage. According to the year of isolation, the viruses in the phylogenetic tree could mainly be clustered into two independent branches labeled A (JS/YZ186/13, JS/YZ562/12, JS/YZ253/12, JS/WJ37/11, JS/JT12/11, JS/YZ640/12, JS/YZ752/12, JS/MH17/14, FJ/SN02/14, JS/YZ177/12, JS/ZJ618/12, HLJ/DLH/11, JS/YZ737/12, JS/YZ150/12, JS/YZ667/12, HB/BD/2011, SD/C9QH/11, JS/YZ687/12, JS/TM59/13 and JS/YZ918) and B (JS/TM71/14, JS/TM58/13, JS/JT95/13 and AH/WB/14). The viruses in branch A which were isolated from 2011–2012 shared a 92%-93.5% sequence identity with the three vaccine strains: SH/F/98, SD/6/96, and GD/SS/94. However, the strains in branch B which were collected from 2013–2015 only had a 90%-92% nucleotide identity with the three vaccine strains (Fig 1a).

**Fig 2:**
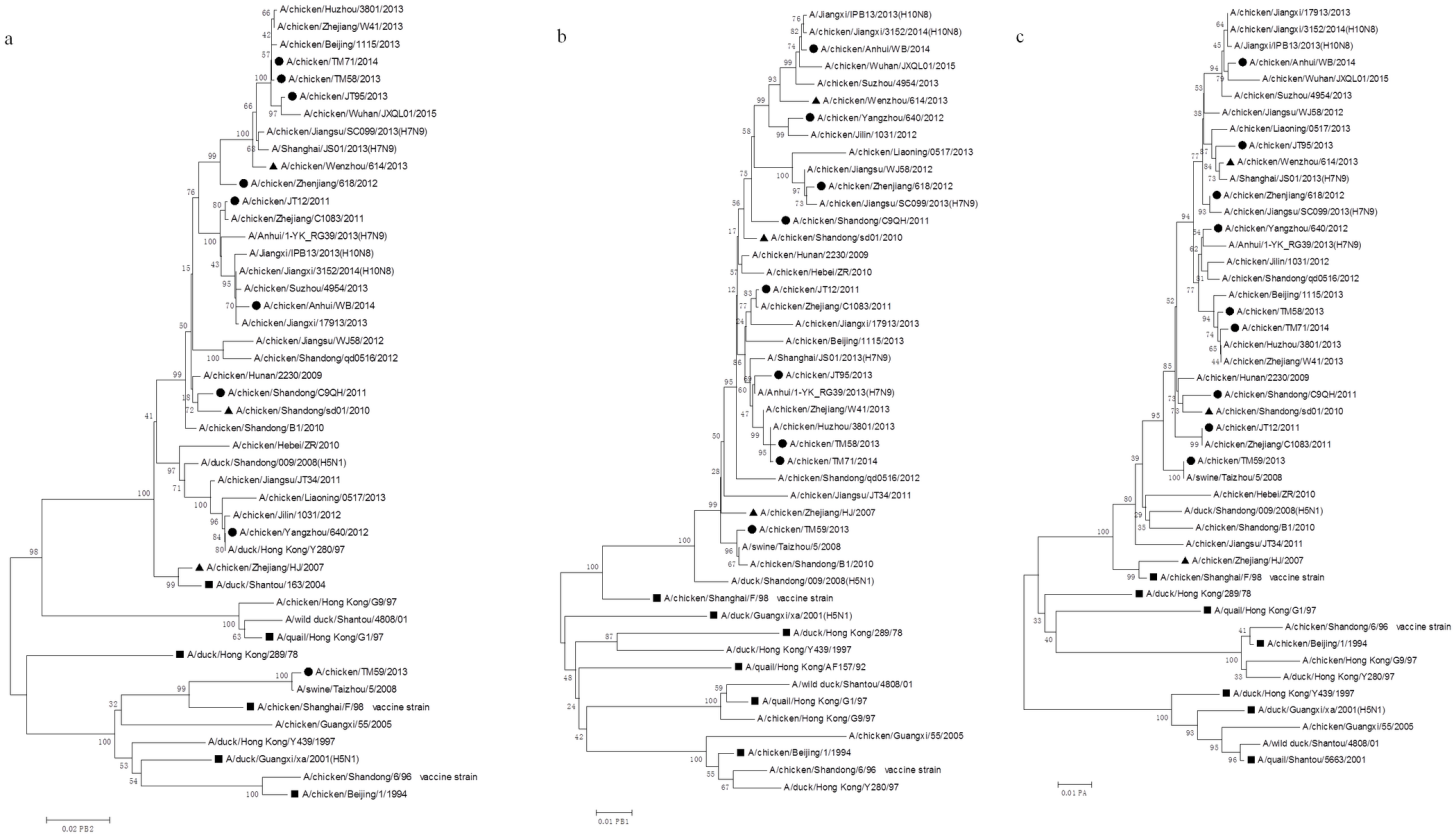
Phylogenetic trees for PB2, PB1 and PA genes collected from mainland China between 2011 and 2014. Solid squares indicate representative strains, solid circles indicate the strains isolated in this study (black circles indicate representative isolates which emerge in the phylogenetic trees for all of the genes except for HA, and white circles indicate the remaining 28 isolates in this study which only emerge in the HA phylogenetic tree), and solid triangles indicate G57 representative strains. Unrooted phylogenetic trees were generated by the distance-based neighbor-joining method using the MEGA6.0 software suite (for the following nucleotide positions: a) PB2, 28–2262; b) PB1, 31–2218; c) PA, 25–2129. The percentage of replicate trees in which the associated taxa clustered together in the bootstrap test with 1000 replicates is shown next to the branches.

**Fig 3:**
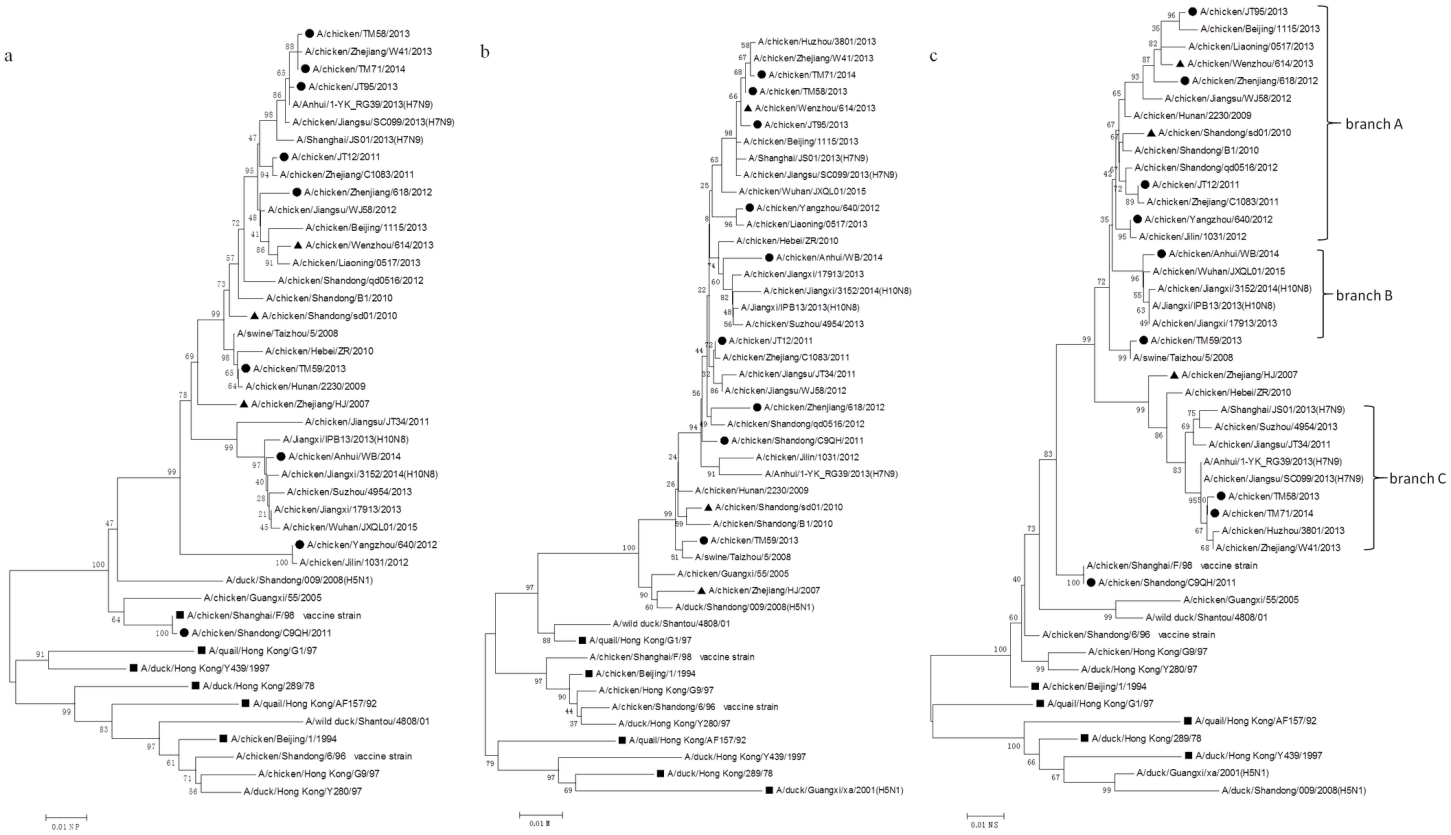
Phylogenetic trees for NP, M and NS genes collected from mainland China between 2011 and 2014. Solid squares indicate representative strains, solid circles indicate the strains isolated in this study (black circles indicate representative isolates which emerge in the phylogenetic trees for all of the genes except for HA, and white circles indicate the remaining 28 isolates in this study which only emerge in the HA phylogenetic tree), and solid triangles indicate G57 representative strains. Unrooted phylogenetic trees were generated by the distance-based neighbor-joining method using the MEGA6.0 software suite (for the following nucleotide positions: a) NP, 35–1456; b) M, 56–960; c) NS, 75–800). The percentage of replicate trees in which the associated taxa clustered together in the bootstrap test with 1000 replicates is shown next to the branches.

Based on the HA phylogenetic analysis, nine isolates (JS/JT12/11, SD/C9QH/11, ZJ/618/12, JS/YZ640/12, JS/TM58/13, JS/TM59/13, JS/JT95/13, JS/TM71/14 and AH/WB/14) were selected, and a phylogenetic analysis of these was performed on the seven genes of interest (NA, PB2, PB1, PA, NP, M, NS). The phylogenetic analysis of the NA nucleotide sequences showed that the viruses in the phylogenetic tree could be mainly grouped into two independent branches labeled A (YZ/640/12, ZJ/618/12, JS/JT12/11 and JS/TM59/13) and B (AH/WB/14, JS/JT95/13, JS/TM58/13 and JS/TM71/14), which were closely related to the year of isolation (Fig 1b). The strains in branch A that were isolated before 2013 shared a 95.2%-96.3% nucleotide identity with the SH/F/98 virus; whereas, the strains in branch B that were isolated after 2013 shared a lower nucleotide homology (94.5%-95%) with the SH/F/98 virus. Phylogenetic trees for the PB2, PB1, PA, NP, M, and NS genes revealed that only the JS/TM59/13 virus belonged to the F/98-like lineage of the PB2 genes, with a 97% nucleotide identity, while the other eight strains and three G57 viruses (ZJ/HJ/07, SD/sd01/10 and WZ/614/13) were grouped into the ST/163-like lineage, with a greater than 98% nucleotide identity (Fig 2a). The PB1, PA, and NP genes from the isolates and three G57 viruses were clustered into the F/98-like lineage (Figs 2b and 2c and 3a–3f). The M genes from the isolates and three G57 viruses were grouped into the G1-like lineage (Fig 3b). The NS genes from the isolates and three G57 viruses were clustered into the BJ/94-like lineage (Fig 3c). Notably, the NS gene phylogenetic tree revealed genetic divergence of the virus gene into three branches labeled A (JS/JT95/13, ZJ/618/12, JS/JT12/11 and JS/YZ640/12), B (AH/WB/14), and C (JS/TM58/13 and JS/TM71/14), which were closely related to the H9N2, H10N8, and H7N9 viruses, respectively (Fig 3c). These results showed that the H9N2 virus internal genes were closely related to those in the human-infected avian influenza viruses, H5N1 and H7N9, and the emerging avian influenza virus, H10N8.

### Antigenic cross-reactivity of the isolates

**Fig 4:**
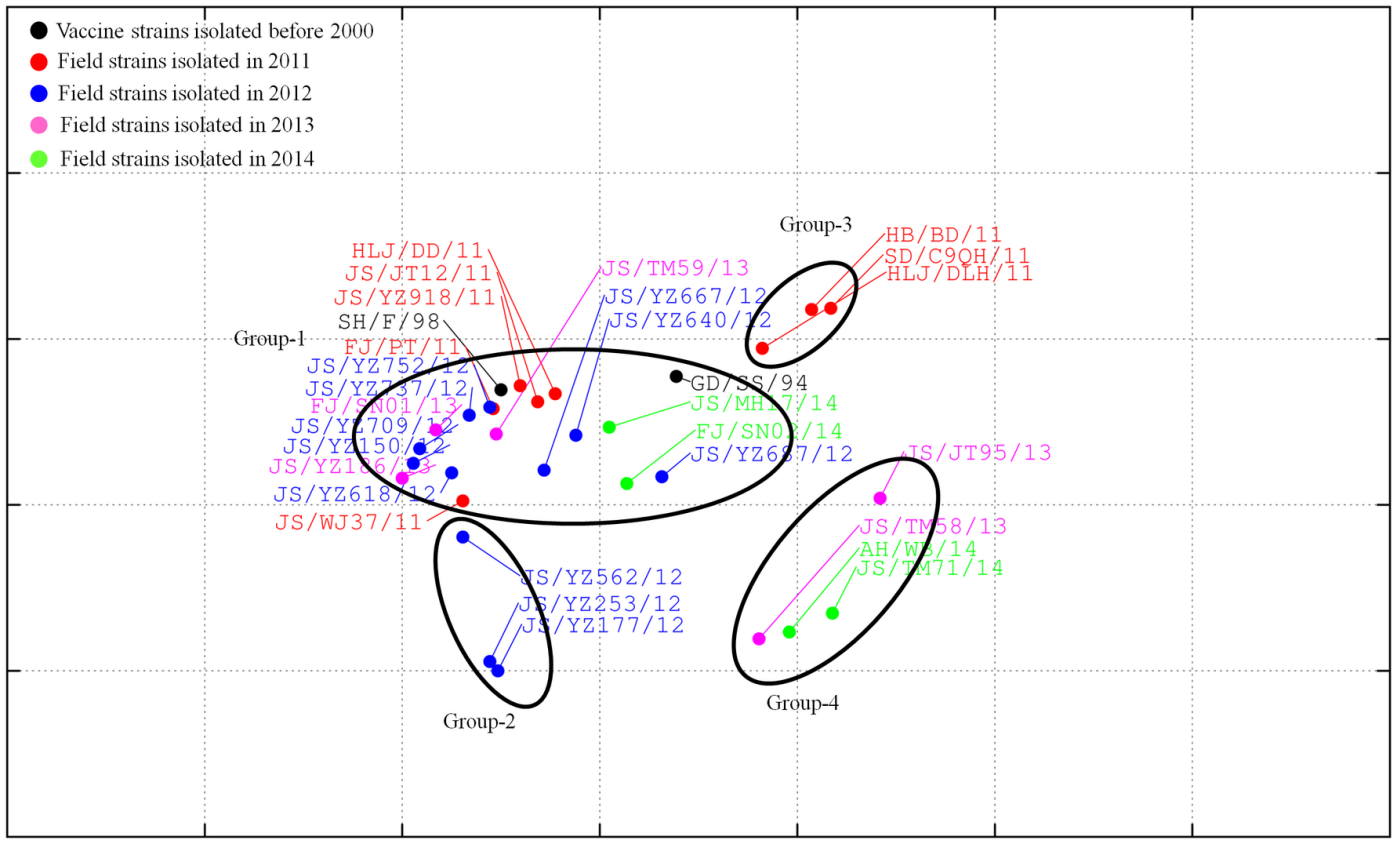
Antigenic map of 28 H9N2 AIV field isolates. Antigenic cartography representations of the HI data generated by using a panel of chicken antisera. In the graph, one grid represents a two-fold change in the HI assay results. Viruses in the same HI group were encircled in an oval.

Results from a reciprocal HI assay demonstrated that antisera against the SD/C9QH/11, JS/TM58/13, JS/JT95/13, or AH/WB/14 strains had low reactivity with most of the isolates and the two vaccine strains, SH/F/98 and GD/SS/94. The two vaccine strains and most of the isolates reacted well with the antisera against JS/TM71/14 or JS/TM59/13 (Table 2). The antiserum against JS/JT12/11 only has reactivity against the vaccine strain SH/F/98 (Table 2). Further AntigenMap analysis showed that all 28 field isolates and the two vaccine strains, SH/F/98 and GD/SS/94, were divided into five independent antigenic groups 1–5 (Fig 4). Group-1 included the vaccine strain SH/F/98 and 16 isolates (HLJ/DD/11, JS/JT12/11, JS/YZ918/11, FJ/PT/11, JS/WJ37/11, JS/YZ752/12, JS/YZ737/12, JS/YZ709/12, JS/YZ150/12, JS/YZ618/12, JS/YZ667/12, JS/YZ640/12, JS/YZ186/13, FJ/SN01/13, JS/TM59/13 and JS/YZ/562). Group-2 consisted of the vaccine strains GD/SS/94, HLJ/DLH/1, JS/YZ687/12, JS/MH17/14, and FJ/SN02/14. Group-3 included HB/BD/11 and SD/C9QH/11. Group-4 consisted of JS/JT95/13, JS/TM58/13, AH/WB/14, and JS/TM71/14. Group-5 included JS/YZ177/12 and JS/YZ253/12. The antigenic distances between Group-4 or Group-5 strains and vaccine strains (SH/F/98 and GD/SS/94) were bigger than those between Group-3 strains and vaccine strains (SH/F/98 and GD/SS/94). Group-3, Group-4, or Group-5 strains had an approximately 2-to 4-fold decrease of HI titers against the antisera compared to the vaccine strains (SH/F/98 and GD/SS/94). The Group-3, Group-4, and Group-5 strains were antigenically distinct from the vaccine strains, SH/F/98 and GD/SS/94. Since Group-3 strains were isolated in 2011, Group-5 strains were isolated in 2012, and Group-4 strains were isolated in 2013 or 2014, the antigenicity of the isolates in 2011–2014 were drifted.

**Table 2:** Cross-reactivity between H9N2 influenza viruses in hemagglutination inhibition assays.

H9N2 viruses*	Antibody titers with antisera to influenza viruses										
	GD/SS//94	SH/F/98	SD/C9QH/11	JS/JT12/11	JS/YZ640/12	JS/YZ618/12	JS/TM59/13	JS/TM58/13	JS/JT95/13	AH/WB/14	JS/TM71/14
GD/SS/94	160	1280	<10	20	20	640	2560	40	<10	40	2560
SH/F/98	160	2560	<10	5120	40	1280	5120	20	<10	40	5120
HLJ/DD/11	80	640	10	640	80	1280	5120	40	40	160	5120
FJ/PT/11	80	2560	20	320	320	640	5120	40	40	160	5120
JS/JT12/11	80	320	<10	320	160	640	5120	40	<10	160	5120
HB/BD/11	10	320	<10	40	40	80	640	40	<10	20	320
SD/C9QH/11	20	<10	5120	80	<10	10	<10	<10	<10	<10	10
JS/YZ918/11	320	1280	<10	640	320	5120	5120	160	20	160	320
HLJ/DLH/11	160	320	5120	<10	<10	<10	<10	<10	<10	<10	20
JS/WJ37/11	160	2560	10	1280	320	2560	5120	160	160	1280	5120
JS/YZ150/12	160	1280	2560	640	320	5120	5120	40	20	160	5120
JS/YZ177/12	2560	1280	320	5120	5120	2560	5120	2560	5120	5120	5120
JS/YZ253/12	160	5120	<10	640	5120	5120	5120	5120	5120	5120	1280
JS/YZ562/12	160	320	20	320	320	2560	5120	320	20	640	5120
JS/YZ618/12	80	1280	10	320	160	5120	5120	80	20	640	5120
JS/YZ640/12	40	640	5120	160	160	640	2560	10	<10	80	5120
JS/YZ667/12	160	80	5120	40	640	2560	10240	160	160	320	5120
JS/YZ687/12	640	1280	1280	80	320	640	320	320	320	320	<10
JS/YZ709/12	640	2560	10	640	320	5120	5120	160	80	320	5120
JS/YZ737/12	160	1280	<10	640	160	2560	5120	80	10	320	640
JS/YZ752/12	160	2560	<10	160	160	1280	5120	40	20	160	1280
JS/TM59/13	160	640	160	160	160	1280	5120	80	20	80	5120
FJ/SN01/13	160	1280	10	640	160	2560	5120	40	<10	80	5120
JS/YZ186/13	320	2560	10	2560	2560	5120	5120	80	320	160	5120
JS/TM58/13	20	320	20	10	80	80	1280	640	5120	5120	5120
JS/JT95/13	20	160	<10	10	<10	80	640	80	640	80	640
JS/TM71/14	320	320	20	10	40	40	320	320	1280	5120	5120
AH/WB/14	40	320	40	20	160	80	1280	640	320	2560	5120
FJ/SN02/14	320	640	80	160	160	<10	5120	80	10	320	2560
JS/MH17/14	80	2560	<10	80	80	2560	2560	40	10	160	5120

*Abbreviations: HLJ, Heilongjiang; FJ, Fujian; JS, Jiangsu; HB, Hebei; SD, Shandong; ZJ, Zhejiang; AH, Anhui; BJ, Beijing; and GD, Guangdong.

### Molecular analysis

**Fig 5:**
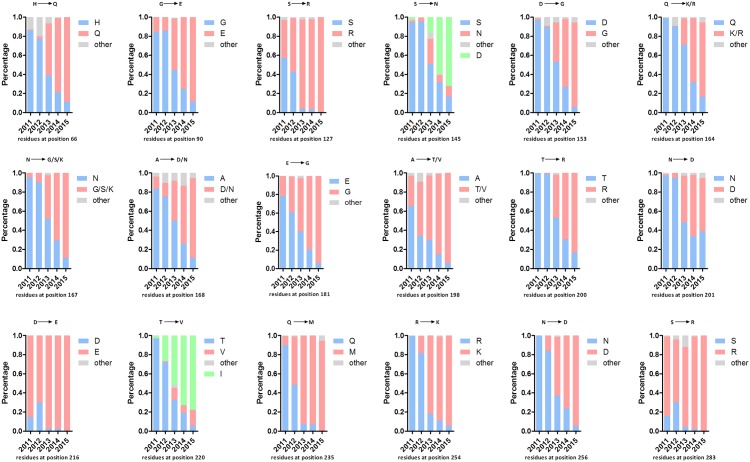
The tendency of mutations at the antigenic residues from 28 isolates from 2010–2015. The mutation levels of each of these amino acid positions with high mutation rates, including H66Q, G90E, S127R, S145N/D, D153G, Q164K/R, N167G/S/K, A168D/N, E181G, A198T/V, T200R, N201D, T220V/I, Q235M, R254K and N256D, and S183N, from the 28 isolates were quantified by comparison with approximately 1081 H9N2 HA sequences deposited in GenBank from 2010 to 2015. The three commercial vaccine strains SH/F/98, SD/6/96, and GD/SS/94 were used as controls. Blue indicates the same amino acid in the three commercial vaccine strains; pink indicates the same mutation in the 28 isolates; gray indicates other mutations, and green indicates that the predominant mutation is different in the 28 isolates.

**Fig 6:**
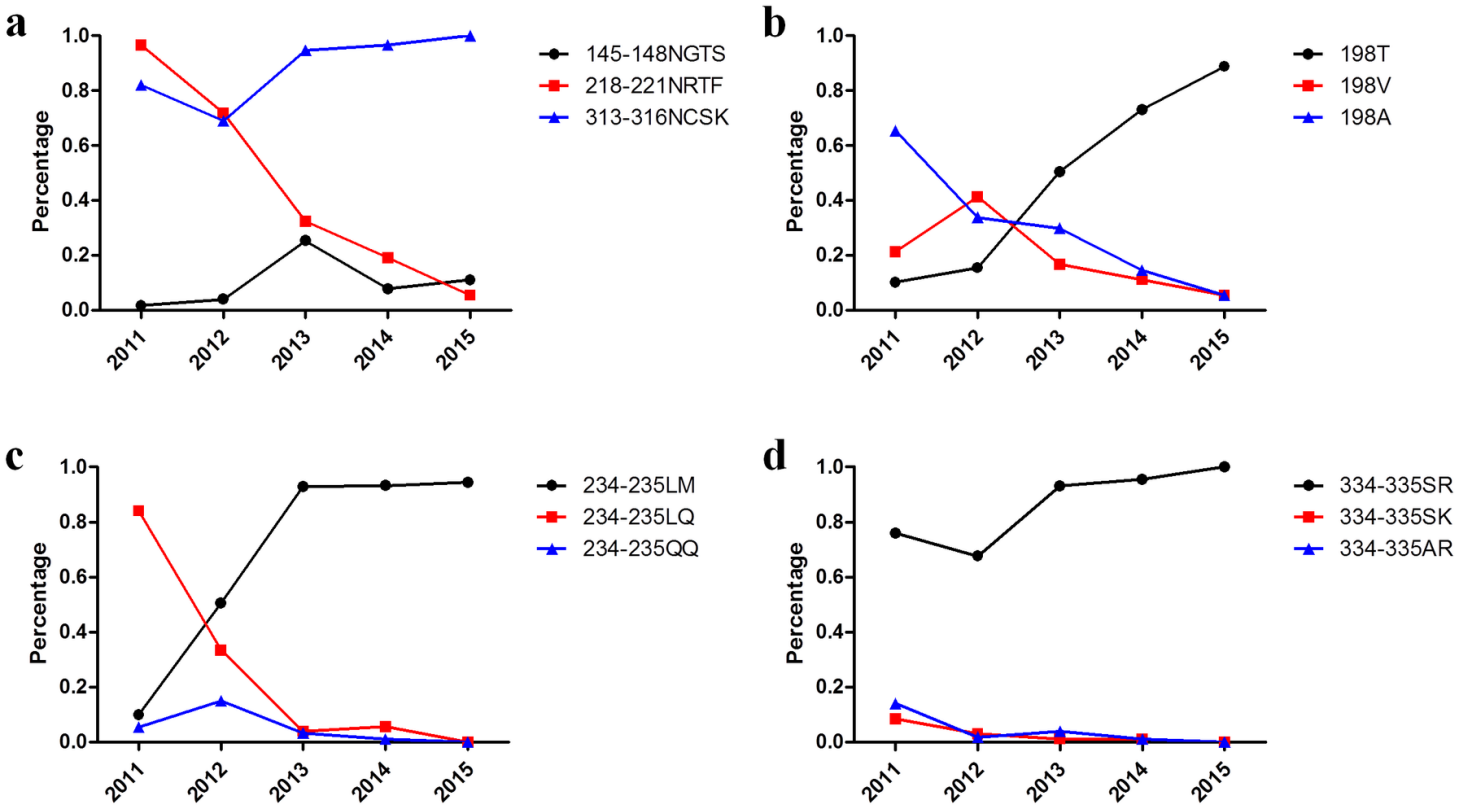
Amino acid changes at key sites on the HA protein from H9N2 viruses from 2010 to 2015. a) The percentage of three potential glycosylation sites, including 145NGTS148, 218NRTF221, and 313NCSK316, was quantified by comparison with approximately 1100 H9N2 HA sequences deposited in GenBank from 2010 to 2015. b) The percentage of mutations at position 198 was quantified by comparison with approximately 1100 H9N2 HA sequences deposited in GenBank from 2010 to 2015. c) The percentage of mutations at positions 234–235 at the right-edge receptor-binding pocket were quantified by comparison with approximately 1100 H9N2 HA sequences deposited in GenBank from 2010 to 2015. d) The percentage of mutations at position 334–335 in the cleavage sites were quantified by comparison with approximately 1100 H9N2 HA sequences deposited in GenBank from 2010 to 2015.

The mutations for each of the 18 reported antigenic residues were analyzed by comparison with 1081 full-length H9N2 HA sequences deposited in GenBank (date to Jan 1, 2016). In comparison to three commercial vaccine strains (SH/F/98, SD/6/96, and GD/SS/94), the 18 antigenic residues in the HA protein of the isolates were changed significantly from 2011–2014, including H66Q, G90E, S127R, S145N, D153G, Q164K/R, N167G/S/K, A168D/N, E181G, A198T/V, T200R, N201D, D216E, T220I, Q235M, R254K, N256D, and S283R (Fig 5). In addition, the antigenic residue mutations S145N and T220V, only appeared in moderate isolates, while the mutations S145D and T220I, were dominant mutations in the published sequences in GenBank (Fig 5). The PNLGSs in HA from the 28 isolates and three vaccine strains were predicted. The results showed that between 7 and 9 PNLGSs were found in these strains, while the FJ/SN/14 virus possessed 10 PNLGSs. PNLGSs at positions 29, 82, 141, 298, 305, 492, and 551 were conserved. Except for positions 82 and 141, these positions were located in the HA stem domain. Non-conserved PNLGSs at positions 145, 196, 218, and 313 were located in the HA globular domain. Specifically, position 313 was found in HA from 28 isolates but not found in the three vaccine strains. The JS/TM71/14, AH/WB/14, JS/TM58/13, FJ/SN/14, JS/JT95/13, and JS/TM59/13 viruses possessed a PNLGS at position 145. Only the JS/YZ253/12 virus possessed a PNLGS at position 196. Seventeen isolates and two vaccine strains possessed a PNLGS at position 218 (Table 3). In this study, a PNLGS at position 145 appeared in the isolates collected from 2013–2014, while a PNLGS at position 218 appeared mainly from 2011–2012. Additionally, the JS/MH17/14 virus possessed a PNLGS at position 218, and the FJ/SN02/14 virus possessed PNLGSs at positions 196 and 218. Furthermore, the PNLGSs at positions 145, 218, and 313 were quantified by comparison with the 1081 full-length H9N2 HA sequences deposited in GenBank (date to Jan 1, 2016). The sequences from three commercial vaccine strains (SH/F/98, SD/6/96, and GD/SS/94) were used as controls. The results showed that the amino acid sequence of NGTS at positions 145–148 (145NGTS148) appeared but only in 2013. The amino acid sequence, 218NRTF221, was dominant from 2011–2012 but began to decrease annually since 2011. The amino acid sequence, 313NCSK316, was only predominant in recent years (Fig 6a). Interestingly, most of the published sequences in GenBank possessed eight PNLGSs (positions 29, 82, 141, 298, 305, 313, 492, and 551) from 2011–2015. These results indicated that changes in the PNLGSs were associated with the year of isolation and that the eight PNLGSs in the H9N2 virus appeared to be relatively stable.

**Table 3:** Potential glycosylation sites of HA amino acid sequences in H9N2 isolates and vaccine strains.

Virus	Potential N-GLy Position										
	The globular domain in HA^a^					The stalk domain in HA					
	82^b^	141	145	196	218	29	298	305	313	492	551
GD/SS/94	+ ^c^	+	-^d^	-	+	+	+	+	-	+	+
SD/6/96	+	+	-	-		+	+	+	-	+	+
SH/F/98	+	+	-	-	+	+	+	+	-	+	+
HLJ/DD/11	+	+	-	-	+	+	+	+	+	+	+
FJ/PT/11	+	+	-	-		+	+	+	+	+	+
JS/JT12/11	+	+	-	-	+	+	+	+	+	+	+
HB/BD/11	+	+	-	-	+	+	+	+	+	+	+
SD/C9QH/11	+	+	-	-	-	+	+	+	+	+	+
JS/YZ918/11	+	+	-	-	+	+	+	+	+	+	+
HLJ/DLH/11	+	+	-	-	+	+	+	+	+	+	+
JS/WJ37/11	+	+	-	-	-	+	+	+	+	+	+
JS/YZ150/12	+	+	-	-	+	+	+	+	+	+	+
JS/YZ177/12	+	+	-		+	+	+	+	+	+	+
JS/YZ253/12	+	+	-	+	-	+	+	+	+	+	+
JS/YZ562/12	+	+	-	-	-	+	+	+	+	+	+
JS/YZ618/12	+	+	-	-	+	+	+	+	+	+	+
JS/YZ640/12	+	+	-	-	+	+	+	+	+	+	+
JS/YZ667/12	+	+	-	-	+	+	+	+	+	+	+
JS/YZ687/12	+	+	-	-	+	+	+	+	+	+	+
JS/YZ709/12	+	+	-	-		+	+	+	+	+	+
JS/YZ737/12	+	+	-	-	+	+	+	+	+	+	+
JS/YZ752/12	+	+	-	-	+	+	+	+	+	+	+
JS/TM59/13	+	+	+	-		+	+	+	+	+	+
FJ/SN01/13	+	+	-	-	+	+	+	+	+	+	+
JS/YZ186/13	+	+		-	-	+	+	+	+	+	+
JS/TM58/13	+	+	+	-	-	+	+	+	+	+	+
JS/JT95/13	+	+	+	-	-	+	+	+	+	+	+
JS/TM71/14	+	+	+	-	-	+	+	+	+	+	+
AH/WB/14	+	+	+	-	-	+	+	+	+	+	+
FJ/SN02/14	+	+	+	-	+	+	+	+	+	+	+
JS/MH17/14	+	+	-	-	+	+	+	+	+	+	+

^a^ The location of potential glycosylation sites in the HA protein.

^b^ Position numbers for the H9 HA amino acid residues.

^c^ Plus signs (+) indicate identical sites.

^d^ Dashes (–) indicate the absence of potential glycosylation sites.

Compared with the three vaccine strains, the receptor-binding sites (RBS) with amino acids Y109, W161, T163, A191, L202, and Y203 were conserved among the 28 isolates, though a mutation occurred at position A198. Among the 28 isolates, 11 strains possessed an A198V mutation, and 8 strains possessed an A198T mutation. The 198T position began to be dominant in 2013, and the proportion of 198V or 198A decreased yearly since 2012 or 2011, respectively (Fig 6b). The amino acid sequence, 146GTSKA150, in the left edge of the receptor binding pocket was conserved among 28 isolates; however, compared with the amino acid sequence, 232NGQQGR237, in the right edge of the receptor binding pocket in the three vaccine strains, the amino acid sequence, 232NGLQGR237, was found in 10 isolates, and the amino acid sequence, 232NGLMGR237, was observed in 18 isolates. The proportion of 234LQ235 sequences decreased rapidly since 2011; whereas, 234LM235 became predominant since 2013 (Fig 6c). Additionally, the 333PSRSSR↓GLF341 cleavage site (H9 numbering) was observed in 23 field isolates, and 333PSKSSR↓GLF341 was observed in five isolates. The 334SR335 sequence was predominant in published sequences from 2011–2015 (Fig 6 and Table 4).

**Table 4:** The key amino acid residues of HA amino acid sequences in H9N2 isolates and vaccine strains.

Virus	The cleavage sites	Left-edge of receptor–binding pocket	Receptor-binding sites							Right-edge of receptor–binding pocket
	333–341	146–150	109	161	163	191	198	202	203	232–237
GD/SS/94	PAGSSR↓GLF	GTSKA	Y	W	T	N	A	L	Y	NGQQGR
SH/F/98	PARSSR↓GLF	GTSKA	Y	W	T	N	A	L	Y	NGQQGR
SD/6/96	PARSSR↓GLF	GTSKA	Y	W	T	N	A	L	Y	NGQQGR
HLJ/DD/11	PSKSSRGLF	GTSKA	Y	W	T	N	T	L	Y	NGLQGR
FJ/PT/11	PSRSSR↓GLF	GTSKA	Y	W	T	N	V	L	Y	NGLQGR
JS/JT12/11	PSKSSRGLF	GTSKA	Y	W	T	N	V	L	Y	NGLMGR
HB/BD/11	PSKSSRGLF	GTSKA	Y	W	T	N	T	L	Y	NGLQGR
SD/C9QH/11	PSRSSR↓GLF	GTSKA	Y	W	T	N	A	L	Y	NGLQGR
JS/YZ918/11	PSRSSR↓GLF	GTSKA	Y	W	T	N	V	L	Y	NGLQGR
HLJ/DLH/11	PSRSSR↓GLF	GTSKA	Y	W	T	N	T	L	Y	NGLMGR
JS/WJ37/11	PSRSSR↓GLF	GTSKA	Y	W	T	N	V	L	Y	NGLMGR
JS/YZ150/12	PSKSSRGLF	GTSKA	Y	W	T	N	A	L	Y	NGLQGR
JS/YZ177/12	PSRSSR↓GLF	GTSKA	Y	W	T	N	A	L	Y	NGLMGR
JS/YZ253/12	PSRSSR↓GLF	GTSKA	Y	W	T	N	T	L	Y	NGLMGR
JS/YZ562/12	PSRSSR↓GLF	GTSKA	Y	W	T	N	T	L	Y	NGLMGR
JS/YZ618/12	PSRSSR↓GLF	GTSKA	Y	W	T	N	T	L	Y	NGLMGR
JS/YZ640/12	PSRSSR↓GLF	GTSKA	Y	W	T	N	V	L	Y	NGLMGR
JS/YZ667/12	PSKSSRGLF	GTSKA	Y	W	T	N	A	L	Y	NGLQGR
JS/YZ687/12	PSRSSR↓GLF	GTSKA	Y	W	T	N	A	L	Y	NGLQGR
JS/YZ709/12	PSRSSR↓GLF	GTSKA	Y	W	T	N	V	L	Y	NGLMGR
JS/YZ737/12	PSRSSR↓GLF	GTSKA	Y	W	T	N	A	L	Y	NGLMGR
JS/YZ752/12	PSRSSR↓GLF	GTSKA	Y	W	T	N	A	L	Y	NGLMGR
JS/TM59/13	PSRSSR↓GLF	GTSKA	Y	W	T	N	V	L	Y	NGLMGR
FJ/SN01/13	PSRSSR↓GLF	GTSKA	Y	W	T	N	V	L	Y	NGLQGR
JS/YZ186/13	PSRSSR↓GLF	GTSKA	Y	W	T	N	V	L	Y	NGLMGR
JS/TM58/13	PSRSSR↓GLF	GTSKA	Y	W	T	N	T	L	Y	NGLMGR
JS/JT95/13	PSRSSR↓GLF	GTSKA	Y	W	T	N	T	L	Y	NGLMGR
JS/TM71/14	PSRSSR↓GLF	GTSKA	Y	W	T	N	T	L	Y	NGLMGR
AH/WB/14	PSRSSR↓GLF	GTSKA	Y	W	T	N	T	L	Y	NGLMGR
FJ/SN02/14	PSRSSR↓GLF	GTSKA	Y	W	T	N	V	L	Y	NGLMGR
JS/MH17/14	PSRSSR↓GLF	GTSKA	Y	W	T	N	V	L	Y	NGLMGR

The hemadsorbing sites (HBS’s, 366–373, 399–403, and 431–433, N2 numbering), active center (140–157), and antigenic determinants (153, 197–199, 328–336, 339–347, 367–370, 400–403, and 431–434) in the NA protein from 9 representative isolates in the two branches were also analyzed [29]. All the sequences from NA in the branch B isolates had mutations at the HBS’s positions 368, 369, and 401–402 (S/R368N, D/G369N/S, and DS401-402EN/DD, respectively) compared with the branch A isolates, which did not have. For the active center, branch A isolates and GD/SS/96 possessed isoleucine at position 153 (I153), while branch B isolates and SH/F/98 contained threonine at position 153 (T153). For the antigenic determinants in NA, D328N, T434P, I153T, KSD/KSG367-369RNN/KNS, and DS401-402EN/DD were observed in both branch A and B isolates; position 153 was also in the NA protein active center (Table 5).

**Table 5:** Molecular characteristics of NA amino acid sequences in H9N2 isolates and vaccine strains.

Virus	Hemadsorbing sites (HBS, N2 numbering)			Active center	Antigenic determinants						
	366–373	399–403	431–433	140–157	153	197–199	328–336	339–347	367–370	400–403	431–434
SD/6/96	IKEDTRSG	DSDNW	PQE	LKNKHSNGTTHDRIPHRT	I	DDK	NDDSSSSSN	DPNNERGAP	KEDT	SDNW	PQET
GD/SS/94	IKEDLRSG	DSDNW	PQE	LKNKHSNGTTHDRIPHRT	I	DDK	NDDNSSSSN	DPNNERGAP	KEDL	SDNW	PQET
SH/F/98	IKKDSRSG	DSDNW	PQE	LKNKHSNGTTHDRTPHRT	T	DDK	NDDSSSSSN	DPNNERGAP	KKDS	SDNW	PQET
SD/C9QH/11	IKSDSRSG	DSDSW	PQE	LKNKHSNGTTHDRIPHRT	I	DDK	DDDSSSSSN	DPNNERGAS	KSDS	SDSW	PQET
JS/JT12/11	IKSDSRSG	DSDSW	PQE	LNNKHSNGTTHDRIPHRT	I	DDK	DDDSSSSSN	DPNNERGAP	KSDS	SDSW	PQET
JS/YZ618/12	IKSGSRSG	DSDSW	PQE	LKNKHSNGTTHDRIPHRT	I	DDK	DDDSSSSSN	DPNNERGAP	KSGS	SDSW	PQET
JS/YZ640/12	IKSDSRSG	DSDSW	PQE	LKNKHSNGTTHDRIPHRT	I	DDK	DDDSFSSSN	DPNNERGAP	KSDS	SDSW	PQET
JS/JT95/13	IRNNSRSG	DSENW	PQE	LKNNHSNGTTHDRTPHRT	T	DDK	NDDSSSSSN	DPNNERGAP	RNNS	SENW	PQEP
JS/TM58/13	IRNNSRSG	DSENW	PQE	LKNNHSNGTTHDRTPHRT	T	DDK	NDDSSSSSN	DPNNERGAP	RNNS	SENW	PQEP
JS/TM59/13	IKRDSRSG	DSDSW	PQE	LKNKHSNGTTHDRIPHRT	I	DDK	DDDSSSSSN	DPNNERGAP	KRDS	SDSW	PQET
JS/TM71/14	IRNNSRSG	DSENW	PQE	LKNNHSNGTTHDRTPHRT	T	DDK	NDDSSSSSN	DPNNERGAP	RNNS	SENW	PQEP
AH/WB/14	IKNSSRSG	DSDDW	PQE	LKNKHSNGTTHDRTPHRT	T	DDK	NDDSSSSSN	DPNNERGAP	KNSS	SDDW	PQEP

Some critical residues in internal genes contributing pathogenicity or infectivity were analyzed, and the results showed that 627E and 701D in PB2 were observed among nine isolates (701N in PB2 was observed only in the virus JS/TM59/13), which are characteristics of low-pathogenic AIVs. In contrast, D253N and Q591K in PB2 were not observed, which could increase AIV pathogenicity [30]. 588V in PB2 was observed in the JS/TM59/13 virus, which could increase the AIV pathogenicity in mammals [31]. In this study, 95K, 224N, and 242N in the M1 protein were observed among 9 isolates, 37A in the M1 protein was observed in eight isolates, and 21G in the M2 protein was observed in 9 isolates, all of which could increase AIV infections [32]. 227K in the NS protein was observed in nine isolates, which also improves the pathogenicity of virus [33]. The nine representative isolates all contained amantadine-resistance mutations at position 13 (proline) in the PB1 protein, position 15 (isoleucine) in the M1 protein, position 55 (phenylalanine) in the M2 protein, and adamantine-resistance mutation at positions 31 (asparagine) in the M2 protein [14, 34, 35]. Both HA 381K and PA 627L in one H9N2 AIV were found to be important for airborne transmissibility among chickens [36], and all strains, except for the SH/F/98 and FJ/SN02/14 strains, possessed both HA 381K and PA 627L. These results from the molecular analysis showed that the field isolates had evolved in the direction of stronger infectivity, pathogenicity, drug resistance, and transmission.

### Replication and transmission of H9N2 influenza virus isolates in chickens

**Fig 7:**
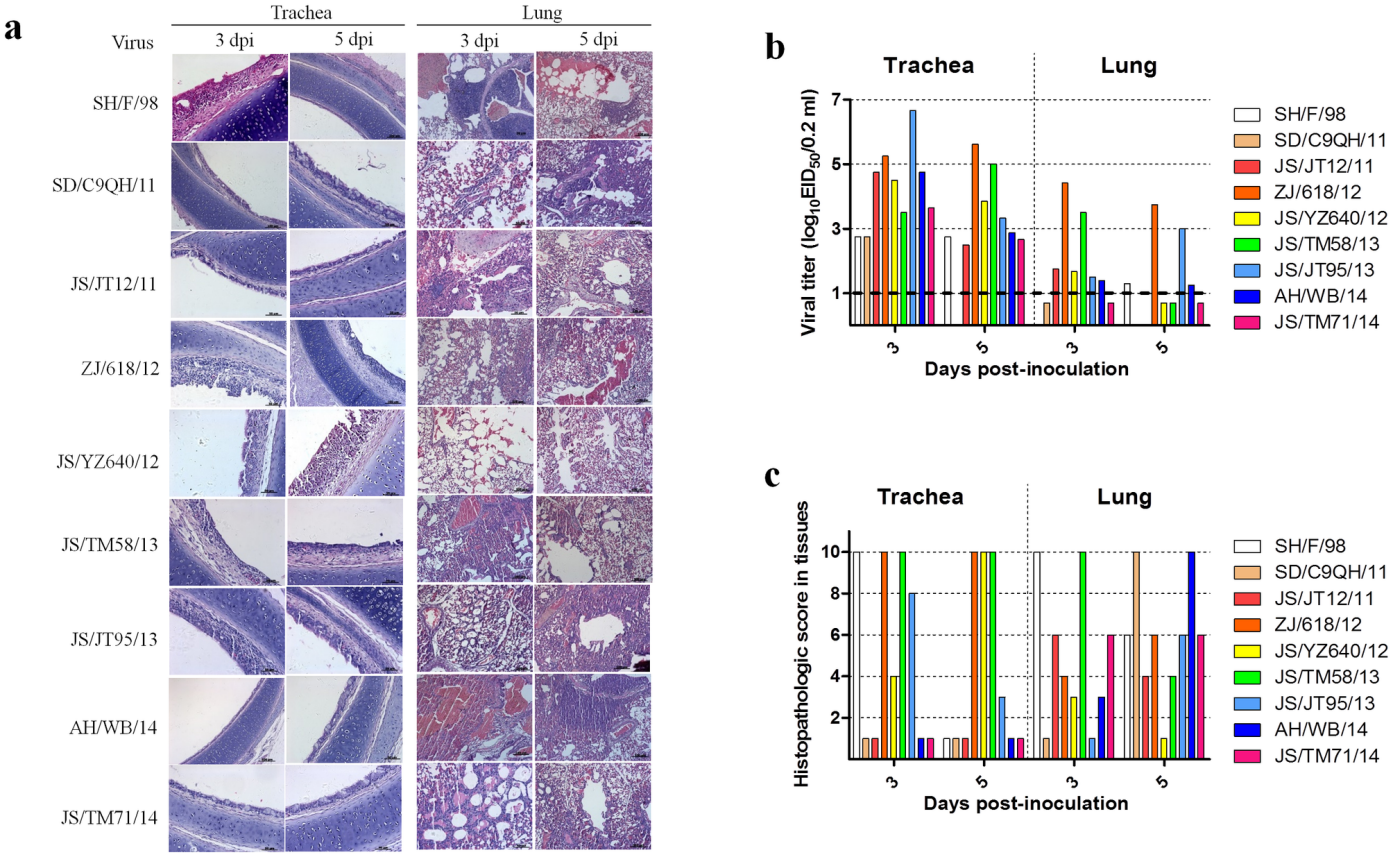
Histopathological changes and virus titers in lungs and tracheae of infected SPF chickens. a) Histopathology of representative infected SPF chickens. Lungs and tracheae were collected at 4 dpi and fixed in 10% formalin, embedded in paraffin, and sectioned. b) Infection of the isolates in chickens. We inoculated groups of chickens intranasally and via the conjunctiva with 10^6^ EID_50_ of the virus. Tracheae and lungs were harvested on 3 and 5 dpi. Viral titers for the tracheal and lung homogenates were determined by endpoint titration in SPF-embryonated chicken eggs. Each patterned bar represents the viral titers from an individual chicken. The black-dashed horizontal line indicates the lower limit of detection. c) Tissue sections were inspected, and histopathological changes were scored as follows. For the tracheae, 0: normal; 1: congestion; 2: cilia loss; 3: little inflammatory cell infiltration; and 7: a lot of inflammatory cell infiltration. For the lungs, 0: normal; 1: congestion; 2: hemorrhage; 3: inflammatory cell infiltration in the bronchial submucosa; and 7: a lot of inflammatory cell infiltration in the bronchial submucosa and alveolus. Average values for three birds are shown. Data are representative of three independent experiments.

To investigate the replication and transmission of H9N2 isolates in chickens, EID_50_ counts from oropharyngeal and cloacal swabs, tracheal and lung homogenates, and tracheal and lung histological specimens were conducted on 3 or 5 dpi. The EID_50_ results from the tracheal and lung homogenates on 3 and 5 dpi showed that all isolates replicated better in tracheae than in lungs. In particular, the JS/JT12/11, ZJ/618/12, JS/YZ640/12, JS/TM58/13, and JS/JT95/13 strains reached higher EID_50_ on 3 and 5 dpi than other strains in the tracheae. The AH/WB/14 and JS/TM71/14 strains reached lower EID_50_ on 5 dpi in the tracheas than on 3 dpi, while no SD/C9QH/11 virus was detected on 5 dpi in the trachea. Only the ZJ/618/12 strain was detected in lung homogenates on 3 and 5 dpi, and the JS/TM58/13 or JS/JT95/13 viruses were detected in lung homogenates on 3 dpi or 5 dpi, respectively, while little or no virus was detected in the lung homogenates on 3 and 5 dpi in the groups infected with the rest of the isolates (Fig 7b). These results showed that the isolates could replicate better in the upper respiratory tract than in the lower respiratory tract. The results from the viral pathogenicity testing in chickens showed that different degrees of lesions were observed in the lungs and tracheae of chickens infected with the isolates. Severe lesions were still observed in trachea infected with the ZJ/618/12, JS/YZ640/12, and JS/TM58/13 strains on 5 dpi, while more severe lesions were observed in tracheae infected with the JS/JT12/11, SD/C9QH/11, ZJ/618/12, JS/JT95/13, JS/TM71/13, and AH/WB/14 strains on 3 dpi than on 5 dpi. The isolates, except for JS/TM58/13, all possess different tissue tropism in the trachea or lungs. Interestingly, although the EID_50_ for most of the isolates from the lungs was low, lesions in the lungs were severe on 3 and 5 dpi (Fig 7a and 7c). The results from the airborne transmission experiments showed distinct airborne transmission among isolates, which may be related to a better ability to shed viruses in the trachea than in the cloaca. The exposed chickens in the JS/JT12/11, ZJ/618/12, JS/YZ640/12, JS/JT95/13, and JS/TM71/14 groups did not shed viruses in the cloaca, and the chickens in the other exposed groups only shed limited viruses. Overall, compared to the trachea, the exposed chickens shed limited or no viruses in the cloaca (Table 6).

**Table 6:** Airborne transmission study of 9 representative isolates. The number of chickens shedding/total number of chickens.

**A**																
**Strains**	SD/C9QH/11 group				JS/JT12/11 group				JS/YZ618/12 group				JS/YZ640/12 group			
	Trachea		Cloacal		Trachea		Cloacal		Trachea		Cloacal		Trachea		Cloacal	
**Virus shedding on day**	3	5	3	5	3	5	3	5	3	5	3	5	3	5	3	5
**Inoculated**	3/3	2/3	0/3	0/3	3/3	3/3	2/3	0/3	3/3	3/3	1/3	2/3	3/3	3/3	2/3	1/3
**Direct contact**	3/3	2/3	1/3	1/3	3/3	2/2	1/3	0/2	2/3	3/3	1/3	1/3	0/1	1/1	1/1	1/1
**Airborne contact**	2/3	3/3	0/3	1/3	1/3	0/3	0/3	0/3	1/3	0/3	0/3	0/3	2/3	2/3	0/3	0/3
**B**																
**Strains**	JS/TM58/13 group				JS/JT95/13 group				JS/TM71/14 group				AH/WB/14 group			
	Trachea		Cloacal		Trachea		Cloacal		Trachea		Cloacal		Trachea		Cloacal	
**Virus shedding on day**	3	5	3	5	3	5	3	5	3	5	3	5	3	5	3	5
**Inoculated**	3/3	3/3	2/3	1/3	3/3	1/3	1/3	0/3	3/3	3/3	1/3	0/3	3/3	3/3	1/3	2/3
**Direct contact**	3/3	2/3	0/3	1/3	2/2	2/2	1/2	1/2	3/3	3/3	1/3	1/3	1/3	1/3	0/3	1/3
**Airborne contact**	1/3	2/3	0/3	1/3	1/3	1/3	0/3	0/3	1/3	0/3	0/3	0/3	1/3	0/3	2/3	0/3

## Discussion

H9N2 subtype avian influenza viruses have caused great economic losses to the poultry industry. The H9N2 virus is recognized as a donor for internal genes to other AIV subtypes, including the H5N1, H7N9, and H10N8 viruses, which can infect humans, causing a potential threat to public health. The H9N2 virus contains a negative, single-stranded RNA molecule that is susceptible to mutation and reassortment during the replication process, and its surface and internal proteins are divided into distinct lineages [26]. Based on HA, the H9N2 AIVs in Eurasia include the following three lineages: BJ/94-like, G1-like, and Y439/97-like. BJ/94-like and G1-like viruses have become the predominant strains in mainland China since the mid-1990s [37]. However, from 2010–2013, a fittest genotype (G57) containing a PB2 gene from dk/ST/04, which is clustered closely to an H7N7 human isolate, A/Netherlands/219/2003 [26], an M gene from the quail origin qa/HK/G1/97, which was closely related to the emerging H7N9 virus [16, 32], an HA gene from ck/JS/00, and the remaining genes from SH/F/98 emerged with changed antigenicity and improved adaptability in chickens [16]. Currently, the G57 virus is the predominant strain in China since 2013 [16].

In this study, 28 H9N2 strains were isolated from live poultry markets in China, including Jiangsu, Anhui, Heilongjiang, and Fujian, from 2011 to 2014. Three G57-type H9 viruses isolated in different years (ZJ/HJ/07, SD/sd01/10, and WZ/614/13) and some field strains isolated in recent years were selected for phylogenetic analysis. The HA segment of the 28 isolates belonged to G57-like viruses. Additionally, the locations of all the strains in the phylogenetic tree are closely related to the year of isolation, and the later the isolation, the farther they are located from the original G57 ZJ/HJ/07 strain in the phylogenetic tree. Specifically, the NA gene has gradually evolved into an undefined lineage. In comparison to other lineages of each gene, the fittest and most advantaged one of each gene may help H9N2 viruses to survive easily in hosts. Additionally, our findings also showed that 16 antigenic residues, two PNLGSs, and one RBS residue changed significantly from 2011–2013, which might be related to the emergence of the G57 lineage. It is generally believed that the RSSR sequence at the HA cleavage site is a characteristic of low-pathogenic AIVs [38]. In this study, we found that the KSSR sequence at the HA cleavage site is also characteristic of low-pathogenic AIVs. The R or K residue at position 335, both of which are alkaline amino acids, may be involved in HA cleavage. Changes in the HA receptor-binding site and its nearby potential glycosylation sites also affect the binding of the virus to host cells and therefore the pathogenicity of the influenza viruses [39]. Although some PNLGSs in isolates potentially have HA sequences increased or decreased from 2011–2013, most published sequences still possessed eight PNLGSs, suggesting that the eight glycosylation sites may be more stable in current H9N2 viruses. The changes in the PNLGSs are closely related to the year of isolation, which is similar to the antigenic residues. Monitoring PNLGSs sites and antigenic residues in circulating viral isolates may facilitate the prediction of mutants with epidemic potential.

The virulence, infectivity, and transmission of the H9N2 virus are affected by multiple factors, particularly genetic factors. In this study, most isolates possessed residues in critical sites that increase the infectivity and transmission. Subsequently, animal experiments showed that the isolates caused different degrees of histopathological changes concerning host tissue tropism and better replication in the upper respiratory tract. Better airborne transmission and replication in the host can create an opportunity for H9N2 viruses to survive easily.

Currently, H9N2 viruses are circulating in China without an effective vaccination strategy. H9N2 viruses escape selection pressure from virus-host interactions by evolution, which may have directly resulted in the emergence of an epidemic G57 virus. To understand the evolution of the H9N2 virus from 2011–2013 when the G57 virus became predominant, we performed a molecular analysis of H9N2 isolates in this study. From 2011–2013 changes in antigenic residues, PNLGSs, and some critical residues might have contributed to the increased H9N2 virus survival and evolution. These changes might help the G57 virus to become the predominant strain and could potentially cause enhanced infectivity and transmission by the circulating H9N2 virus.

## Conclusions

In summary, our findings suggest that the H9N2 virus surface glycoproteins have undergone evolution and variation from 2011–2014, which might be related to the G57 virus outbreaks and emergence since 2013. The H9N2 virus internal genes were closely related to those from the human-infected avian influenza viruses H5N1, H7N9, and the emerging influenza virus H10N8. In particular, some NS genes from H9N2 viruses were closely related to the H10N8 and H7N9 viruses, respectively.
